# Lactoferrin amyloid presenting as a mural nodule in a pancreatic cystic lesion prompting pancreatoduodenectomy: a case report

**DOI:** 10.1186/s12876-021-01641-8

**Published:** 2021-02-12

**Authors:** Katherine A. Baugh, Svetang Desai, George Van Buren 2nd, William E. Fisher, Carlos A. Farinas, Sadhna Dhingra

**Affiliations:** 1 Department of Surgery, Baylor College of Medicine, Houston, TX USA; 2 Gastroenterology Consultants, Houston, TX USA; 3 Department of Radiology, Baylor College of Medicine, Houston, TX USA; 4 Department of Pathology and Immunology, Baylor College of Medicine, 1 Baylor Plaza, MS BCM315, Houston, TX 77030 USA

**Keywords:** Pancreas, Amyloid, Mucinous cyst, Mural nodule, Case report

## Abstract

**Background:**

Amyloid deposition in pancreas is rare. Lactoferrin amyloid deposition has not been reported in pancreas, till date. Presence of enhancing mural nodule in a cyst on imaging is a worrisome feature for malignancy, and warrants surgical resection in a surgically fit candidate, as per Fukuoka guidelines for management of cystic lesions in pancreas.

**Case report:**

We report a case of localized amyloidosis presenting as a mural nodule in a 1.6 cm cyst located in the head of pancreas, which led to pancreatoduodenectomy in a 69 year old woman. Histological evaluation revealed a simple mucinous cyst with localized lactoferrin amyloid deposition corresponding to the mural nodule identified on imaging.

**Conclusions:**

We report the first case of localized lactoferrin amyloid deposition in pancreas that presented as a mural nodule in a cystic lesion and prompted pancreatoduodenectomy. This unique case illustrates that on rare occasion mural nodule in a cyst can be benign. It adds amyloid deposition to the differential diagnosis of mural nodules in pancreatic cystic lesions seen on imaging.

## Background

Amyloidosis arises from disordered protein metabolism in which normally soluble proteins undergo conformational changes causing insolubility and extracellular deposition [1]. Amyloid deposition can occur in almost every organ in the body with particularly common occurrence in the liver, kidneys, and skin [1–3]. Amyloid deposition in pancreas is rare [4–8]. Systemic amyloidosis can uncommonly involve the pancreas, and is incidentally detected on autopsy as this does not lead to functional abnormalities of exocrine or endocrine function [9]. Localized amyloid deposition in the islets of Langerhans is more frequent in the pancreas in patients with diabetes mellitus. The type of amyloid fibril in this setting is islet amyloid polypeptide (IAPP). The only pancreatic tumor associated with amyloidosis is Insulin expressing pancreatic neuroendocrine tumor, which is rarely associated with IAPP-type amyloidosis [8].

Lactoferrin is an iron binding protein present in the granules of polymorphonuclear leukocytes (PML) [10–13]. Upon activation of PMLs, lactoferrin is excreted with the intention of binding free iron that is needed for bacterial growth. Lactoferrin as a precursor protein of localized amyloidosis is rare and has only been reported as isolated cases in conjunctiva, cornea, seminal vesicles and bronchus [14–17]. Amyloid lactoferrin has never been reported in the pancreas to the best of our knowledge. Herein, we report a novel case of localized lactoferrin amyloidosis manifesting as a mural nodule in a 1.6 cm pancreatic cystic lesion, detected on endoscopic ultrasound (EUS) imaging. Presence of lymphadenopathy on imaging and “atypical cells” on fine needle aspiration cytology of nodule categorized the lesion as a “mucinous cyst with worrisome features” [18], and prompted pancreatoduodenectomy.

## Case presentation

**Fig. 1:**
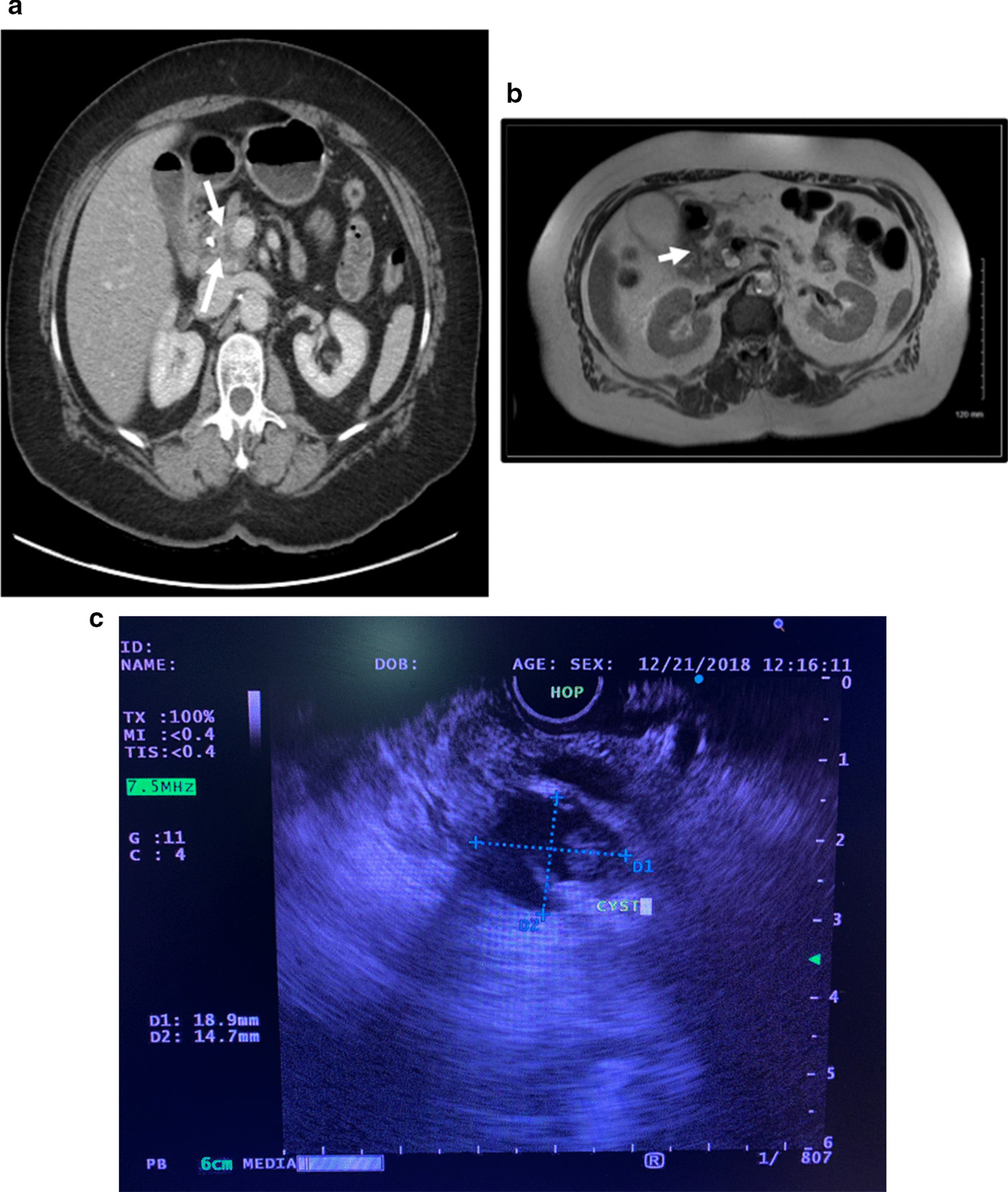
**a** CT scan demonstrating an indeterminate hypodense area in the pancreatic head (arrows) (axial view). **b** MRI scan demonstrating the lobulated cyst with eccentric mural nodule in the head of the pancreas with effacement of the distal common bile duct. Associated atrophy of the body and tail of the pancreas (axial view). **c** EUS showing cyst wall with mural nodule (arrow)

A 69 year old Caucasian female with a past history of ocular melanoma was found to have an incidental 1.6 × 1.6 × 0.7 cm hypodense lesion in the head of the pancreas, on surveillance computed tomographic (CT) scanning of the abdomen (Fig. 1a). In addition, there was a single enlarged porta hepatis lymph node measuring 2.8 × 1 cm. The patient was asymptomatic and without any prior episodes of pancreatitis.

Additional imaging studies were performed to characterize the lesion. Magnetic resonance imaging (MRI) demonstrated the 1.7 × 1.3 cm lobulated lesion with effacement of the distal CBD (Fig. 1b). Endoscopic ultrasound (EUS) confirmed the 1.5 × 1.4 cm hypoechoic lesion suggestive of a cyst, which did not show communication with the pancreatic duct. EUS further demonstrated thickened cyst wall with mural nodule and presence of luminal debris (Fig. 1c). The mural nodule measured 0.8 × 0.7 cm. Vascularity of the nodule on EUS was not assessed. Fine needle aspiration of the cyst revealed turbid, viscous fluid, however the pathology evaluation was inconclusive. Post-EUS, the patient developed right upper quadrant abdominal pain, with post-prandial worsening, and was associated with dark colored urine and acholic stools. Due to concern for extrinsic compression on the CBD, the patient underwent ERCP, which identified a 7 mm single area of stenosis in the CBD that was relieved with placement of a plastic stent. Following stent placement, her blood work and symptoms normalized. She underwent repeat EUS with FNA of mural nodule. The FNA cytologic evaluation revealed atypical cells. Clinically, the cyst was considered to be an intraductal papillary mucinous neoplasm or mucinous cystic neoplasm. Considering the worrisome features of lymphadenopathy on imaging studies and atypical cells on FNA cytology of mural nodule, a decision was made to resect the lesion. Pancreas protocol CT scan performed prior to surgery showed a vague hypodense area in the pancreatic head, likely collapse of the cyst following aspiration, and a decompressed biliary tree. Pancreatic duct was not dilated, however, there was distal pancreas atrophy. A pylorus preserving pancreaticoduodenectomy was performed.

**Fig. 2:**
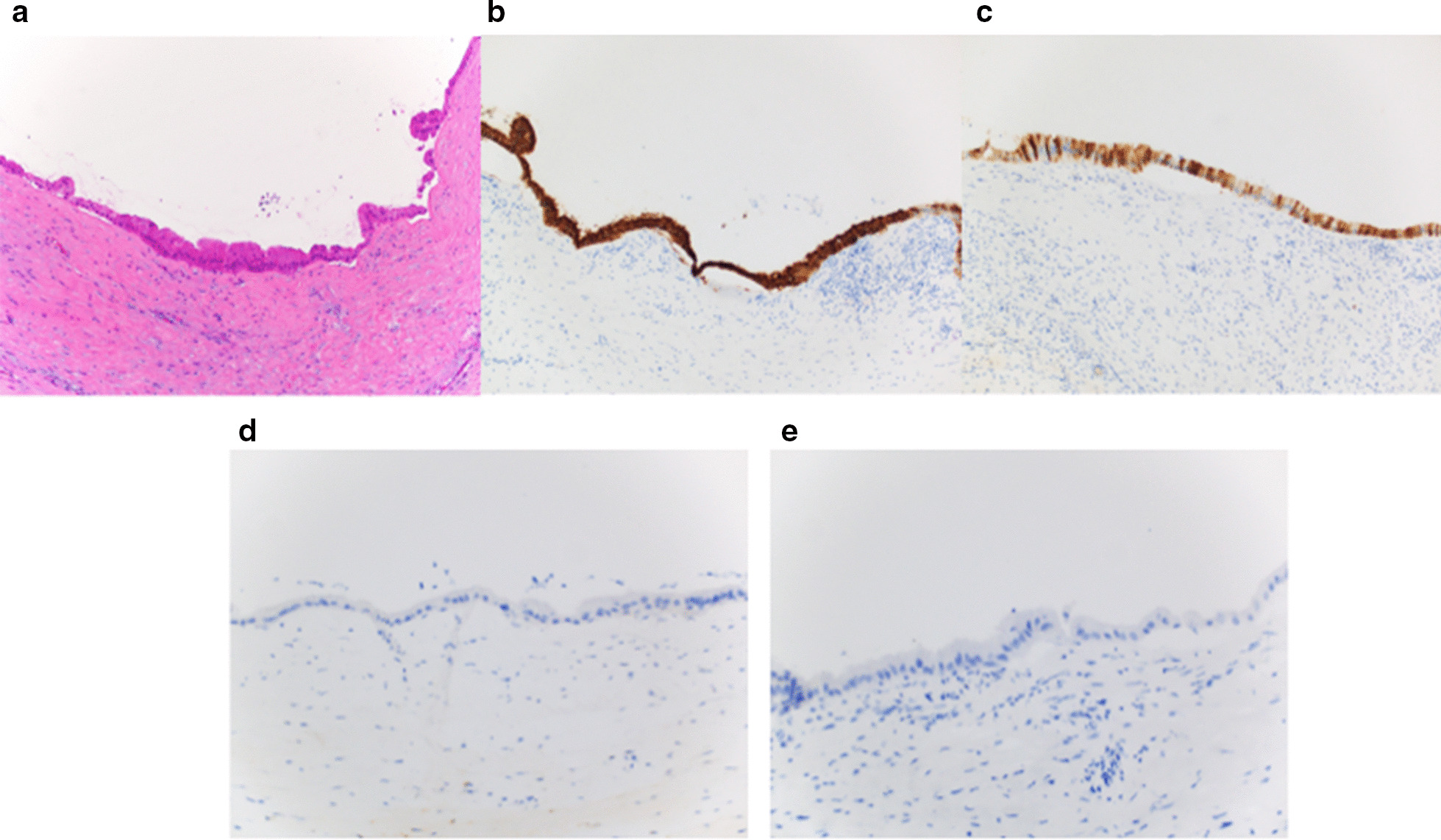
**a** Cyst wall lined by simple mucinous lining epithelium. Hematoxylin and Eosin stain. × 200. **b**–**e** Cyst wall epithelium positive for MUC 5AC and MUC 6 (**b**, **c**) and negative for MUC1 and MUC 2 (**d**, **e**). Immunostains for MUC5AC, MUC6, MUC1, MUC 2. × 200

**Fig. 3:**
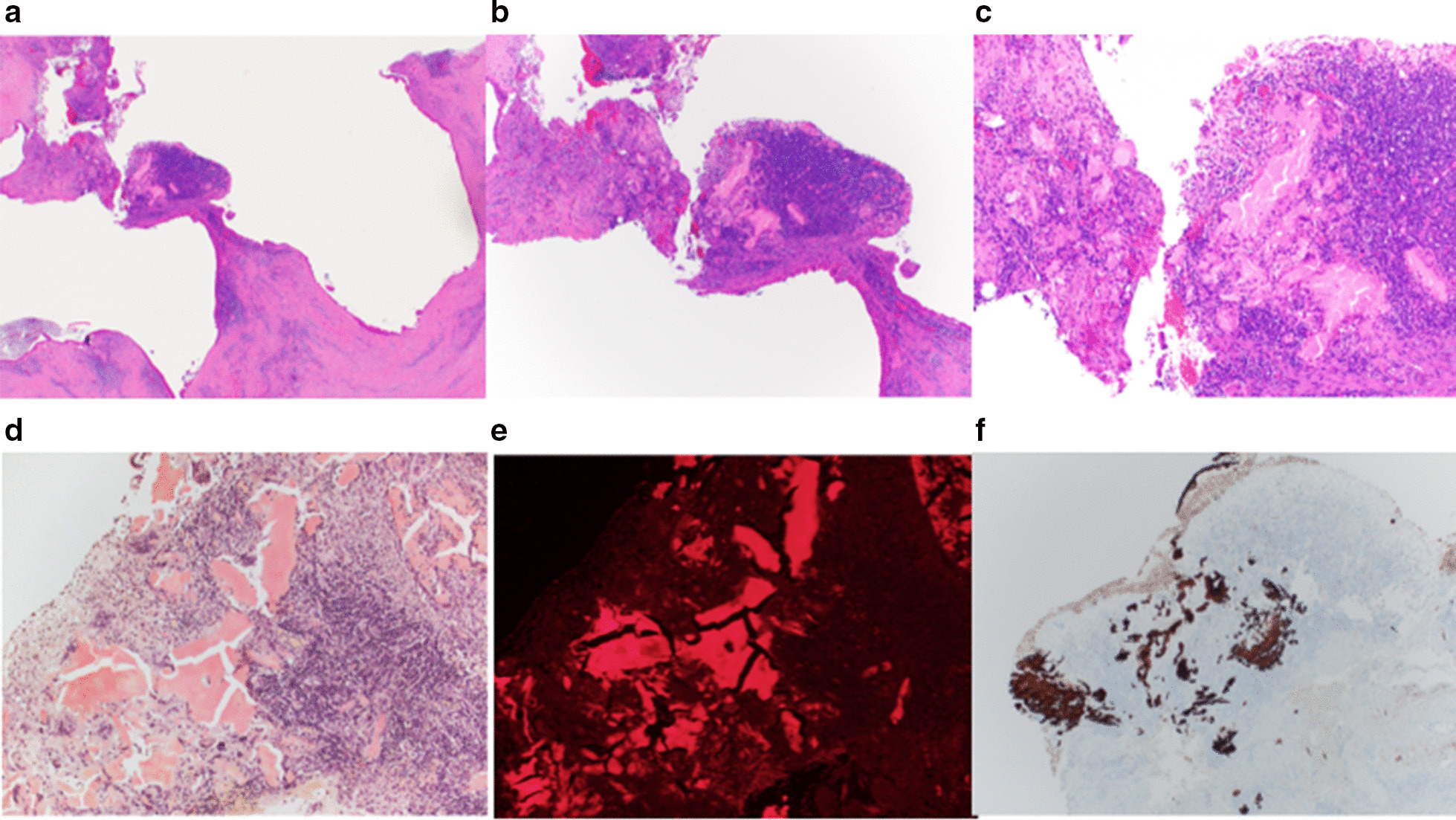
**a** Unilocular cyst with mural nodule (arrow). Hematoxylin and Eosin stain. × 20. **b** Unilocular cyst with mural nodule (arrow). Hematoxylin and Eosin stain. × 40. **c** Mural nodule composed of amorphous eosinophilic material admixed with chronic inflammation. Hematoxylin and Eosin stain. × 100. **d** Amorphous material is congophilic, consistent with amyloid. Congo red stain. × 100. **e** Amyloid deposits with red fluorescence by fluorescence microscopy using Texas red filter. Congo red stain × 100. **f** Immunostain for lactoferrin, positive in amyloid deposits. × 100

Gross evaluation of the pancreas revealed a 1.6 × 1.1 × 1 cm uniloculated cystic lesion in the head of the pancreas. The cyst did not show communication with the main pancreatic duct. The cystic lesion was completely excised and all margins were negative. The entire lesion was submitted for histological evaluation. Microscopic evaluation showed a unilocular cyst lined by gastric type mucinous epithelium (Fig. 2a). No papillary projection or configuration was seen. Immunohistochemical stains showed the cyst wall lining to be diffusely positive for MUC5AC, and focally positive for MUC 6. The mucinous lining was negative for MUC1 and MUC2 (Fig. 2b–e). The histological features were consistent with a simple mucinous cyst. No dysplasia or carcinoma was seen. There was focal thickening in the cyst wall with intraluminal nodular lesion characterized by amorphous eosinophilic congophilic material consistent with amyloid, surrounded by foreign body type giant cell reaction and chronic inflammation composed of lymphocytes and polyclonal plasma cells (Fig. 3a–d). Upon further evaluation of congo red stain, the amyloid deposits showed characteristic apple green birefringence on polarizing microscopy, and red fluorescence on fluorescent microscopy using Texas Red filter (Fig. 3e). This focus correlated with the mural nodule seen on EUS. Laser microdissection (LMD)-liquid chromatography-tandem mass spectroscopy (LC–MS) confirmed the amyloid to be lactoferrin type. In addition, an immunostain for lactoferrin was positive in the amyloid deposits (Fig. 3f). The background pancreas showed mild chronic pancreatitis. No amyloid deposition was noted in the islets of Langerhans, interstitium or blood vessels of pancreas.

Her post-operative course was uneventful apart from a mild wound seroma requiring only local wound care. She has since been seen multiple times in clinic following surgery and has recovered well.

## Discussion and conclusions

Lactoferrin amyloid deposition has never been reported in the pancreas. The case is unique because of unusual presentation of localized amyloidosis as a mural nodule in a 1.6 cm cystic lesion in the head of pancreas, detected by endoscopic ultrasound. As per current international consensus Fukuoka guidelines [18] for management of intraductal papillary mucinous neoplasms of the pancreas, a cyst < 3 cm in size with an enhancing mural nodule on imaging, is considered to be a “cystic lesion with worrisome features”, with a recommendation to proceed with resection in a surgically fit patient without further testing. The premise for this recommendation is that the enhancing mural nodule in the cystic lesion has so far been noted to be an invasive adenocarcinoma developing in mucinous neoplasms. Although we don’t have information about enhancement of mural nodule, ours is a unique case report that shows a mural nodule composed of amyloid lactoferrin deposition and associated giant cell reaction.

The pathogenesis of amyloid lactoferrin in the pancreas remains unclear. This could be secondary to chronic inflammation. Some authors theorize that mutations in lactoferrin cause significant conformational changes prompting the formation of insoluble amyloid fibrils [16, 19]. However, no definitive mechanism has been identified in previous reports of deposits in the cornea, bronchus, and seminal vesicles [14–17]

Diagnosis frequently requires more than just the initial clinical presentation and thus, imaging is usually necessary. In this case, multiple imaging modalities, including CT, MRI, EUS, and ERCP, were utilized. Only few case reports describe imaging findings associated with amyloidosis of the pancreas. Onur et al. reported CT findings of a diffusely hypoechogenic pancreas with associated enlargement and calcifications [4]. Krishna et al. described a patient with amyloid presenting as a hypoechoic solid mass with anechoic spaces on EUS [20]. None of the findings previously described are unique to amyloid deposition and histopathology is integral to diagnosis.

Amyloid deposition will typically present as amorphous eosinophilic acellular waxy deposits on histopathologic evaluation. When stained with congo red stain, the deposits are congophilic, impart apple green birefringence on polarizing microscopy and show red fluorescence on fluorescent microscopy using Texas Red filter [21]. Though presentation and imaging may suggest alternate diagnoses, histopathological confirmation of amyloidosis is usually necessary, as was the case for this patient.

As diagnosis of lactoferrin amyloid deposition in the pancreas is novel there is no current standard of care for its treatment. In this case, surgical resection was recommended due to the initial concerns for malignancy developing in IPMN/MCN as well as presence of clinical symptoms. Following surgery, the patient has undergone standard post-operative follow-up with no further surveillance specific to her diagnosis suggested. Therefore, appropriate treatment and surveillance likely hinges upon the burden of symptoms experienced by the patient, presence or concern for additional pathology, and whether it represents a localized or systemic process.

In conclusion, localized lactoferrin amyloid deposition in the pancreas presenting, as a mural nodule in an asymptomatic pancreatic cystic lesion is novel. Additional features of lymphadenopathy and atypical cells on FNAC of nodule were concerning for a malignancy, which led to surgical resection. Pathologic evaluation revealed localized amyloid deposition, lactoferrin type, limited to the focus of mural thickening in a simple mucinous cyst lined by gastric type mucinous epithelium. No dysplasia or carcinoma was seen. This is a unique case that adds a new clinical differential to the imaging feature of mural nodule in pancreatic cysts.

## Data Availability

Not applicable.

## Abbreviations

IAPP: Islet amyloid polypeptide
PML: Polymorphonuclear leukocytes
EUS: Endoscopic ultrasound
mg/dl: Milligram/deciliter
IU/L: International units/Litre
ALT: Alanine aminotransferase
AST: Aspartate aminotransferase
CBD: Common bile duct
ERCP: Endoscopic retrograde cholangiopancreatography
FNA: Fine needle aspiration
CT: Computed tomography
MUC 5AC: Mucin5AC
MUC6: Mucin6
MUC1: Mucin1
MUC2: Mucin2
LMD: Laser microdissection
LC–MS: Liquid chromatography–tandem mass spectroscopy
MRI: Magnetic resonance imaging
IPMN/MCN: Intraductal papillary mucinous neoplasm/mucinous cystic neoplasm
